# Making MicroED Accessible at a CryoEM Center Through Training and Infrastructure

**DOI:** 10.1063/4.0000793

**Published:** 2025-10-27

**Authors:** Edward T Eng, Christina Zimanyi, Daniel Decato, Jessalyn G Miller, Chun-Hsing Chen

**Affiliations:** 1 New York Structural Biology Center, New York, NY, USA; 2 University of Montana, Missoula, MT, USA; 3 University of North Carolina at Chapel Hill, Chapel Hill, NC, USA

## Abstract

Cryogenic electron microscopy (cryoEM) is powerful technology that may be applied to the structural sciences by leveraging the widely used and directly translatable modalities of single particle analysis and electron tomography. However, integrating micro electron diffraction (microED) into an existing cryoEM facilities present unique challenges including staff and instrument availability, expertise, and standard protocols. At the Simons Electron Microscopy Center (SEMC) housed at the New York Structural Biology Center (NYSBC), we have worked to lower barriers for researchers interested in all cryoEM modalities by implementing targeted cross-training initiatives, and fostering collaborations between microscopists and crystallographers. As a national cryoEM service center, NYSBC is committed to broadening access to structural biological technologies by sharing best practices, facilitating training, and ensuring that these workflows are not limited to specialized groups. As part of this mission, we aim to enable researchers by cross-training them to independently lead their own research projects. Our overall goal is to make all users view microED as a viable and accessible technique within a structural biologist's toolkit and strive to help scientists leverage the resources within their home cryoEM facilities to pursue a variety of research initiatives.

